# Tuberculoma of the Tongue Presenting as Hemimacroglossia

**DOI:** 10.1155/2012/548350

**Published:** 2012-12-19

**Authors:** S. P. S. Yadav, Arpit Agrawal, J. S. Gulia, Sunita Singh, Arsh Gupta, Vineet Panchal

**Affiliations:** ^1^Department of Otolaryngology, Post Graduate Institute of Medical Sciences (PGIMS), Rohtak, Haryana, India; ^2^Department of Pathology, Post Graduate Institute of Medical Sciences (PGIMS), Rohtak, Haryana, India

## Abstract

The underdiagnosis of extrapulmonary tuberculosis lesions, along with an emerging global resistance to antitubercular drugs, warrants an increased awareness of the involvement of *Mycobacterium tuberculosis* in atypical lesions of oral cavity. Tongue is the most common site of oral tuberculosis. We report a rare presentation of lingual tuberculosis in a 65-year-old male, a chronic tobacco chewer, who came to us with swelling of the tongue which apparently looked like hemimacroglossia, leading to the clinical diagnosis of submucosal carcinoma of tongue. Enlargement of tongue is a slow process resulting from gradual invasion and lodging of bacilli in the tongue. Biopsy and histopathological examination revealed tuberculous etiology of the lesion and the patient responded well to antitubercular therapy.

## 1. Introduction

Tuberculosis (TB), an infectious disease is caused by *Mycobacterium tuberculosis*, an acid fast bacillus that is transmitted primarily through the respiratory tract. Tuberculosis affects approximately 8 million people worldwide with relatively high incidence in the Indian subcontinent, South East Asia, and Africa [1]. Tuberculosis chiefly affects the pulmonary system, but it can involve extrapulmonary sites too. Tuberculosis of oral cavity has been generally regarded as a rare entity; it rarely features in the differential diagnosis of oral lesions. Nowadays, oral manifestations of tuberculosis are reappearing alongside many forgotten extrapulmonary lesions, as a consequence of the outbreak of drug-resistant TB along with the emergence of acquired immune deficiency syndrome (AIDS). Inspite of increasing incidence in extrapulmonary TB, it is still an underdiagnosed entity [2]. This is partly due to involvement of atypical sites with rare clinical presentations. We report a case of lingual tuberculosis in a 65-year-old male who presented with hemimacroglossia, which demonstrates the difficulty in making the diagnosis of extrapulmonary TB and needs a high index of suspicion. 

## 2. Case Report

**Figure 1:**
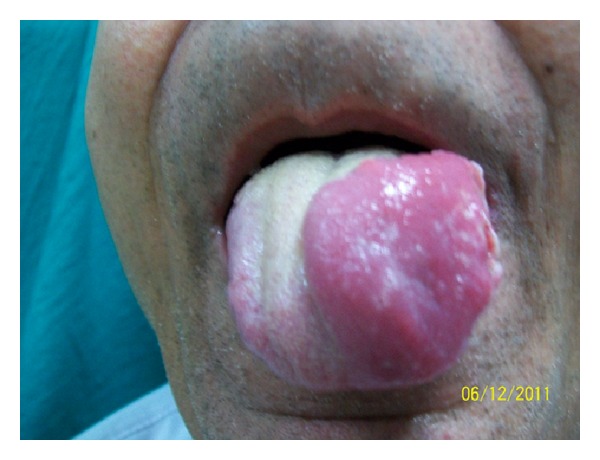
Photograph showing hemimacroglossia.

**Figure 2:**
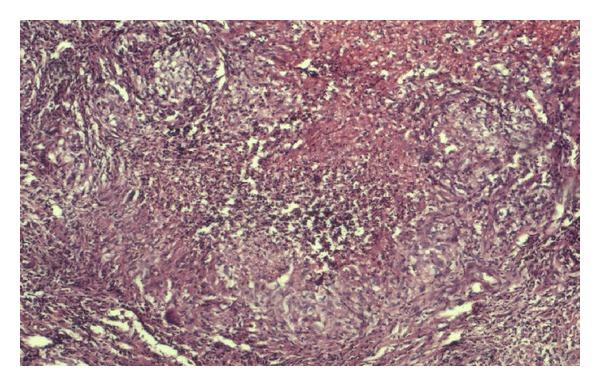
Photomicrograph showing epitheloid cell granulomas with central necrosis (H&E, 100X).

**Figure 3:**
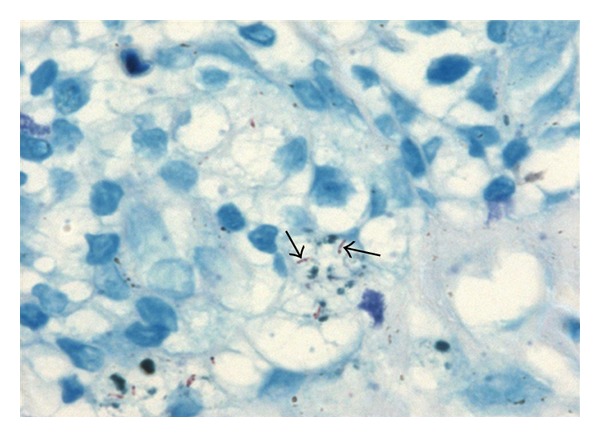
Ziehl-Neelsen stain showing acid-fast bacilli (arrows, 1000X).

A 65-year-old male presented to us with a painful, progressively increasing swelling of the tongue of 8 months duration. There was no history of cough, fever, dysphagia, or weight loss. He was a nonsmoker, but a tobacco chewer for the past 15 years. He was an alcoholic since past 15 years. He was not a known case of diabetes mellitus, but a hypertensive since past 4 years and was on regular treatment for it. On examination, patient was well built with good general condition. Examination of oral cavity revealed an 8 × 6 cm firm swelling over the left side of anterior 2/3rd of tongue, crossing the midline, with smooth surface and well-defined margins. The lesion apparently looked like hemimacroglossia (Figure 1). There was no deviation of tongue, with all movements being normal. No lymphadenopathy was found in the neck. Systemic examination did not reveal any abnormal findings. Clinical suspicion of submucosal carcinoma of tongue was entertained. Complete blood count was within normal limits. ESR was found raised to 35 mm in first hour. A deep punch biopsy from lateral aspect of the lesion was taken, which revealed granulomatous inflammation suggestive of tuberculosis (Figure 2). Acid fast bacilli were found in Ziehl-Neelsen stained sections (Figure 3). A histopathological diagnosis of tuberculosis was made. Mantoux test performed with PPD antigen measured 18 mm after 72 hours reading. ELISA test for HIV was nonreactive. Chest X-ray was within normal limits. With a final diagnosis of primary tuberculoma of tongue, patient was started on antitubercular therapy with a four drug regimen for 6 months. Dramatic improvement was observed within 2 months of therapy. Size of the lesion was significantly reduced, from an apparent hemimacroglossia to a small nodular lesion. After 6 months the patient was completely cured and is on regular followup with no residual or recurrent lesion.

## 3. Discussion

A significant proportion of patients (15–25%) exist in whom active TB is manifested in an extrapulmonary site [3]. Oral cavity constitutes 0.2 to 1.5% of all extrapulmonary tuberculosis sites [4]. The low number of oral infections by *M. tuberculosis* could be due to underreporting [5]. The oral sites most commonly involved are tongue, palate, tonsil, pharynx, and buccal mucosa [6]. The cleansing effect of saliva, relative paucity of lymphoid tissue in the tongue, and the antagonist oral commensals are all reasons for decreased virulence of TB in oral cavity. Tongue is a muscular organ with good vascularity and activity further contributing to the defense against tuberculosis [7]. Morgagni first described the case of lingual tuberculosis in 1761 [8]. Lingual tuberculosis is more frequently diagnosed in immunocompromised patients, males and smokers [9]. Tuberculosis in oral cavity is usually secondary to pulmonary involvement. Pulmonary involvement results in increased concentration of bacteria in the mouth, along with hematogenous or lymphatic transmission of bacilli to secondary sites. Primary TB of tongue is extremely rare [10]. Primary tuberculosis of oral cavity usually occurs following a breach in the oral mucosa as a result of chronic tobacco use, traumatizing dentures, tooth extractions, or poor oral hygiene. Hence, role of trauma cannot be underestimated, as the stratified squamous epithelium of oral cavity normally resists direct penetration of tubercle bacilli. In secondary tuberculosis, the oral manifestations are accompanied by lesion in lung, lymph nodes, or any other site of the body. This can be detected by the usual clinical history and systemic examination. Primary oral tuberculosis may thus present as a diagnostic challenge to the clinician [11]. Clinically, the commonest presentation of primary tuberculosis of tongue is ulcerative lesion along the lateral border of tongue [12]. “Macroglossia” in tuberculosis is extremely rare, which is defined as an enlarged tongue. Enlargement of half of the tongue is termed as “hemimacroglossia” as was observed in the present case. Macroglossia is a clinical presentation which has an extensive list of possible causes such as haemangioma, lymphangioma, Down syndrome, Beckwith Wiedemann syndrome, Hurler syndrome, Becker and Duchenne dystrophies, hypothyroidism, acromegaly, neurofibromatosis, tuberculosis, diabetes mellitus, glossitis, polymyositis, and idiopathic. Severe macroglossia can lead to cosmetic and functional disturbances as in speaking, eating, swallowing, and sleeping. 

Aird has described five pathological types of lesion due to tuberculosis [13].

1. Tubercular ulcers: It is painful and usually develops as a small tubercle which later on softens to form an ulcer. Typically they are shallow, ovoid, with undermined, margins and lined with pale granulation tissue.
2. Tuberculoma: Originates as a lump anywhere in the tongue resembling lingual gumma. The lump in due course of time softens to form an ulcer.
3. Tuberculous fissure: They usually occur on the side of tongue, can be appreciated only by separating their edges.
4. Tubercle papilloma: An overgrowth of the margins of tubercular fissures.
5. Tubercular cold abscess: Due to breakdown of a tuberculoma.

Investigation for an oral TB should almost universally include a tissue biopsy, which has been found to be 88% specific in published case reports [14]. With respect to histopathology, TB is one of the granulomatous inflammatory diseases. Macrophages, lymphocytes, and fibroblasts are among the cells that aggregate to form granuloma, with lymphocytes surrounding the infected macrophages. Tuberculous granulomas also features necrosis in the centre, which to the naked eye has the texture of soft, white cheese, hence termed as caseous necrosis. Multinucleated Langhans giant cells are often present around the periphery of the granuloma. The granuloma prevents dissemination of the mycobacterium and provides a local environment for interaction of cells of the immune system. Bacilli inside the granuloma can become dormant, resulting in latent infection as well. Biopsy analysis is used to rule out systemic diseases such as Wegner's granulomatosis, sarcoidosis, Behcet's disease, Crohn's disease, Melkersson-Rosenthal syndrome, syphilis, and, of course, oral cancers.

History and clinical examination plays a vital role in the diagnosis of oral tuberculosis. However, in our case, the rare presentation of lingual tuberculosis in the form of swelling of the tongue in the form of hemimacroglossia, led us to the clinical misconception. Finally, the diagnosis was established only after a biopsy and histopathological examination which thus emphasizes the role of this gold standard investigation in the diagnosis of such tongue masses. Primary involvement of the tongue in this case could be explained with the history of chronic tobacco chewing, leading on to trauma and subsequent infestation by tubercle bacilli. Such a big lesion has probably resulted from lodging of bacilli in the tongue and gradual invasion into the substance of tongue over the course of many years, although the patient could have noticed it in later stages. If the amount of bacilli is high enough in oral tuberculosis lesion, there is a possibility that more lesions could be induced and/or disseminated to other individuals via saliva. Therefore, no matter whether primary or secondary oral tuberculosis, an early suspicion and timely intervention can lead to favorable outcome in such cases, both for the patient and community.

## 4. Conclusion

 We would like to conclude that though primary tuberculosis of a tongue is a rare entity and such a presentation is even rarer, yet tuberculosis needs to be kept in the differential diagnosis of a tongue mass. Biopsy plays a key role in establishing the diagnosis in such a situation. A meticulous clinical and pathological evaluation of atypical but curable lesion of tongue is being emphasized.
